# Comparison of Clinical Characteristics and Prognosis in Patients with Right- and Left-sided Infective Endocarditis

**DOI:** 10.5041/RMMJ.10338

**Published:** 2019-01-28

**Authors:** Vered Stavi, Evgenia Brandstaetter, Iftach Sagy, Sabina Sapunar, Roman Nevzorov, Carmi Bartal, Leonid Barski

**Affiliations:** 1 Department of Internal Medicine F, Soroka University Medical Center, Beer–Sheva, Israel; 2 Department of Internal Medicine E, Soroka University Medical Center, Beer–Sheva, Israel

**Keywords:** Clinical characteristics, etiology, pathogenesis, prognosis, right-sided endocarditis

## Abstract

**Objective:**

Right-sided endocarditis (RSE) accounts for 5%–10% of all cases of infective endocarditis (IE) and frequently has different etiological, pathogenetic, and clinical presentations compared with left-sided endocarditis (LSE). The aims of this study were to evaluate the epidemiologic and clinical characteristics and prognosis of RSE patients and to compare them with those of LSE patients. This study’s importance relates to the local understanding of RSE and LSE, since Israeli demographics are different compared to the Unites States and Europe with regard to intravenous drug abuse and rheumatic valvular disease prevalence.

**Material and Methods:**

A retrospective cohort study of 215 patients with infective endocarditis was performed. The primary outcome was in-hospital mortality. The secondary outcomes were duration of hospitalization, recurrent hospitalization, recurrent infective endocarditis, and one-year mortality.

**Results:**

Of the 215 patients in the study, 176 had LSE and 39 had RSE. The RSE patients were younger than the LSE patients (48.1±18.9 years versus 61.8±17.0 years, *P*<0.001). The most common pathogen in both groups was *Staphylococcus aureus*, which occurred more in the RSE group (51%) versus the LSE group (19%). In-hospital mortality was lower among patients with RSE (2.6% versus 17%, *P*<0.037).

**Conclusions:**

Our study demonstrated an increasing percentage of RSE compared to LSE among patients with IE. Pacemaker lead infection has become the leading cause of RSE in intravenous drug users (IVDU), although less common in Southern Israel. The etiological and clinical differences between RSE and LSE are noteworthy. Patients with RSE have a better prognosis than those with LSE.

## INTRODUCTION

Right-sided endocarditis (RSE) accounts for 5%–10% of all cases of infective endocarditis (IE) and most frequently involves the tricuspid valve.^1^,^2^ At-risk populations include intravenous drug users (IVDU) and patients who have undergone intravenous catheterization. Other risk factors are alcohol abuse, immunodeficiency, and congenital heart defects.^3^–^7^

Patients with RSE may be classified into three groups: (1) IVDU; (2) cardiac device carriers; and (3) no left-sided endocarditis (LSE), no IVDU, and no cardiac device. Each group can be considered as independent entities due to the relevant epidemiologic, clinical, microbiological, echocardiographic, and prognostic differences among them.^8^

The most common pathogenic cause of RSE is *Staphylococcus aureus*, among both IVDU and the rest of the patient population.^1^,^9^–^13^ Other RSE-causing pathogens are coagulase-negative staphylococci and Gram-negative bacilli; there are also cases with polymicrobial involvement.^9^,^12^,^14^ The pathogenesis of RSE is unclear. Several mechanisms have been proposed: direct injection of the pathogen into the venous blood stream followed by an encounter with the tricuspid valve, immunological mechanisms, and endothelial injury.^15^,^16^

The clinical presentation of RSE often involves fever accompanied by respiratory signs and symptoms secondary to septic emboli to the lungs.^3^,^4^,^17^ Due to this unique clinical presentation the diagnosis of RSE is often delayed.^18^ Hence, a strong clinical suspicion must be present in order to establish a diagnosis.^18^–^20^

The aims of this study were to evaluate the epidemiologic, clinical, and laboratory characteristics of RSE with regard to patients, etiology, and pathogenesis, as compared with LSE, and to evaluate and compare the patient outcomes for RSE versus LSE.

Since Israeli demographics are quite different from those of the Unites States and Europe with regard to intravenous drug abuse and rheumatic valvular disease prevalence, this study has potential importance for the treatment of RSE and LSE patients in Israel.

## MATERIALS AND METHODS

The study focused on IE patients admitted to Soroka University Medical Center (SUMC). The center is the only tertiary hospital for a population of 1.1 million, geographically spread out through an area half the size of the State of Israel.

Institutional Review Board approval was obtained prior initiation of the study. A retrospective cohort study was performed of all adult patients with RSE hospitalized in SUMC between 2003 and 2013, since all medical records were computerized in 2003, making the data easily accessible.

Discharge diagnoses (ICD-9) were used to identify subjects with infective endocarditis according to ICD-9 codes: 112.81, 397, 421.0-421.9, 424, 424.2, 424.9.

All IE cases were reviewed by two investigators (senior physicians in internal medicine) according to the modified Duke criteria for IE diagnosis.^20^

The patients’ demographic characteristics, ICD-9 diagnoses, medications, and clinical and laboratory data were obtained from a comprehensive medical chart review and from the computerized hospital database.

Patients with RSE were compared to patients with LSE. In-hospital mortality (primary outcome), duration of hospitalization, recurrent hospitalization, recurrent infective endocarditis, and one-year mortality (secondary outcomes) were obtained.

Data were expressed as mean ± standard deviation (SD), median ± interquartile range (IQR), or number and percentage. Comparison of RSE and LSE patient characteristics was performed using *t* test, chi-square, and non-parametric tests. Survival curves were calculated by the Kaplan–Meier method, and comparison between patient groups was performed by log-rank test. A two-sided *P* value <0.05 was considered as statistically significant.

## RESULTS

The study included 215 patients diagnosed with IE based on the modified Duke criteria. Of these, 176 had LSE and 39 had RSE.

Patient demographic and clinical characteristics are detailed in Table 1. The majority of patients were male (total of 132 men and 44 women across both groups). The percentage of men and women in both groups was similar. Patients with RSE were younger than patients with LSE (48.1±18.9 years versus 61.8±17.0 years, *P*<0.001).

**Table 1:** Comparison of Epidemiologic, Clinical, and Laboratory Characteristics of Patients with Left- and Right-sided Endocarditis.

Parameters	Right-sided Endocarditis *n*=39	Left-sided Endocarditis *n*=176	*P* value
Age, mean±SD	48.1±18.9	61.8±17.0	<0.001

Male sex, *n* (%)	28 (71.8)	104 (59.1)	0.1

IV drug user, *n* (%)	17 (43.6)	7 (4.0)	<0.001

Endocarditis in the past, *n* (%)	6 (15.4)	5 (2.8)	0.006

Cardiac pacemaker, *n* (%)	8 (20.5)	23 (13.1)	0.3

Inflammatory reaction after cardiac device, prosthetic valve, and graft, *n* (%)	6 (15.4)	64 (36.4)	0.01

Congestive heart failure, *n* (%)	6 (15.4)	27 (15.3)	1

Diabetes mellitus, *n* (%)	7 (17.9)	50 (28.4)	0.2

Malignancy, *n* (%)	8 (20.5)	22 (12.5)	0.5

Chronic pulmonary disease, *n* (%)	1 (2.6)	20 (11.4)	0.1

Connective tissue disease, *n* (%)	none	6 (3.4)	0.5

Chronic liver disease, *n* (%)	7 (17.9)	3 (1.7)	<0.001

Cardiac arrhythmias, *n* (%)	8 (20.8)	68 (38.6)	0.04

Rheumatic heart disease	3 (7.7)	33 (18.8)	0.1

Rheumatoid factor (IU/mL), median (IQR)	17 (11;68)	35 (12;90)	0.09

C-reactive protein (mg/L), median (IQR)	17 (6;32)	7.3 (4.7;11.5)	0.008

C3 (mg%), median (IQR)	116 (76;140)	129 (105;146)	0.4

C4 (mg%), median (IQR)	26 (12;28)	29 (23;35)	0.09

Surgery, *n* (%)	9 (23.1)	47 (26.8)	0.7

**Type of Surgery**			
AVR, *n* (%)	none	16 (9.1)	0.04

MVR, *n* (%)	none	17 (9.7)	0.04

Pacemaker lead extraction, *n* (%)	3 (7.7)	7 (4.0)	0.3

Pacemaker extraction, *n* (%)	1 (2.6)	2 (1.1)	0.4

TVR/tricuspid valve excision, *n* (%)	2 (5.1)	none	0.03

Triple valve surgery (AVR+MVR+TVR), *n* (%)	1 (2.6)	1 (0.6)	0.3

Double valve surgery (AVR+MVR), *n* (%)	none	3 (1.7)	1

Double valve surgery (MVR+TVR), *n* (%)	none	1 (0.6)	1

**Main Cause of Admission**			

Fever, *n* (%)	27 (69.2)	115 (65.3)	0.7

Chest pain, *n* (%)	2 (5.1)	4 (2.3)	0.3

Heart failure, *n* (%)	3 (7.7)	12 (6.8)	0.7

Weakness, anemia, *n* (%)	none	9 (5.1)	0.4

Stroke, *n* (%)	none	7 (4.0)	0.4

Abnormal echocardiography, *n* (%)	none	7 (4.0)	0.4

Infected pacemaker pocket, *n* (%)	2 (5.1)	none	0.03

Weight loss, *n* (%)	none	4 (2.3)	1

Cough and hemoptysis, *n* (%)	1 (2.6)	1 (0.6)	0.3

Other, *n* (%)	4 (10.3)	15 (8.5)	0.8

AVR, aortic valve replacement; IV, intravenous; IQR, interquartile range; MVR, multiple valve replacement; SD, standard deviation; TVR, tricuspid valve replacement.

Among the RSE patients there were more IVDU (43.6% versus 4.0%, *P*<0.001) and more patients with a prior IE episode (15.4% versus 2.8%, *P*= 0.006). More LSE patients suffered from cardiac arrhythmia (38.6% versus 20.8%, *P*<0.04), whereas more RSE patients suffered from chronic liver diseases (17.9% versus 1.7%, *P*<0.001).

There were no statistically significant differences between the two groups in most laboratory parameters, except for an elevated C-reactive protein (CRP) in the RSE group (17 [IQR 6;32] mg/L versus 7.3 [4.7;11.5] mg/L, *P*<0.008).

With regard to the reason for hospital admission, more RSE patients were admitted due to an infected pacemaker pocket (5.1% versus none, *P*=0.03). However, there were more cases of inflammatory reactions after cardiac device, prosthetic valve, and grafts in the LSE group (36.4% versus 15.4%, *P*=0.01).

The etiologic factors for RSE and LSE are presented in Table 2. The most common pathogen in both groups was *Staphylococcus aureus*, which occurred in more than half of the RSE group (51%) as compared to the LSE group (19%).

**Table 2:** Etiologic Factors of Left- and Right-sided Endocarditis.

Etiology	Right-sided Endocarditis *n*=39	Left-sided Endocarditis *n*=176	*P* value
*Staphylococcus aureus*, *n* (%)	20 (51.3)	33 (18.8)	<0.001
Staphylococci, coagulase-negative, *n* (%)	none	24 (13.6)	0.009
Streptococci, viridans, *n* (%)	4 (10.3)	26 (14.8)	0.6
Streptococci, other types, *n* (%)	3 (7.7)	13 (7.4)	1
HACEK group, *n* (%)	none	8 (4.5)	0.3
Gram-negative rods, *n* (%)	2 (5.1)	4 (2.3)	0.3
Enterococcus, *n* (%)	1 (2.6)	23 (13.1)	0.08
Fungi, *n* (%)	1 (2.6)	2 (1.1)	0.4
Pseudomonas, *n* (%)	none	4 (2.3)	1
Brucella, *n* (%)	none	3 (1.7)	1
Q-fever, *n* (%)	none	3 (1.7)	1
Unknown pathogen, culture-negative, *n* (%)	8 (20.5)	30 (17)	0.6

HACEK, *Haemophilus aphrophilus*, *Actinobacillus actinomycetemcomitans*, *Cardiobacterium hominis*, *Eikenella corrodens*, and *Kingella kingae*.

There were no statistically significant differences in the pathologic findings of either group on transesophageal echocardiography (Table 3).

**Table 3:** Echocardiographic Findings on Transesophageal Echocardiography.

Echocardiographic Findings	Right-sided Endocarditis*n*=39	Left-sided Endocarditis*n*=176	*P* value
Vegetation, *n* (%)	23 (59)	99 (56.3)	0.8
Abscess, *n* (%)	1 (2.6)	2 (1.1)	0.4
Mass attached to electrode of the pacemaker, *n* (%)	2 (5.1)	8 (4.5)	1
New severe valvular insufficiency, *n* (%)	1 (2.6)	4 (2.3)	1
Prosthetic valve dehiscence, *n* (%)	none	1 (.6)	1
Absence of findings typical for endocarditis, *n* (%)	12 (30.8)	61 (34.7)	0.7

**Figure 1:**
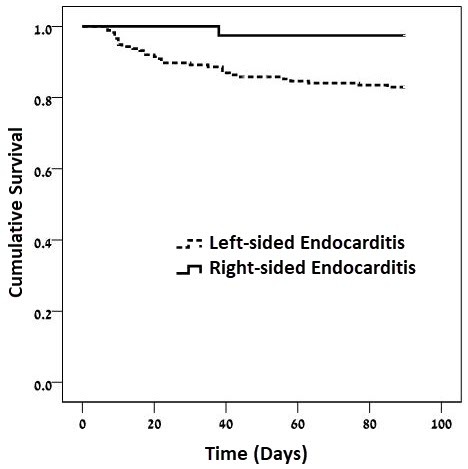
Kaplan–Meier In-hospital Survival Curves Stratified by Endocarditis with Left- and Right-side Involvement Log-rank test *P*=0.023.

**Figure 2:**
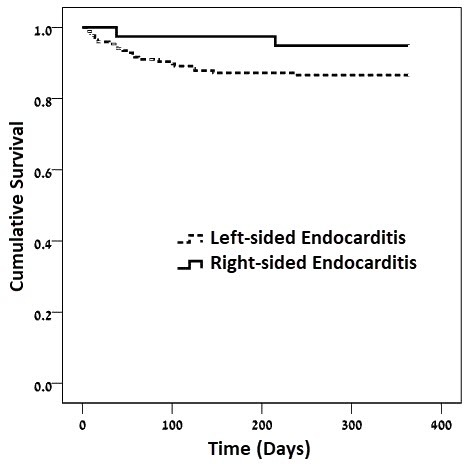
Kaplan–Meier One-year Survival Curves Stratified by Endocarditis with Left- and Right-side Involvement Log-rank test *P*=0.015.

In-hospital mortality rates were lower among patients with RSE (2.6% versus 17%, *P*<0.037), as shown in Figure 1 and Table 4. There were no statistically significant differences in other outcomes between the two groups of patients. One-year survival curves for RSE and LSE are shown in Figure 2.

**Table 4:** Outcomes of Patients with Left- and Right-sided Endocarditis.

Parameters	Right-sided Endocarditis*n*=39	Left-sided Endocarditis*n*=176	*P* value
Recurrences of endocarditis, *n* (%)	4 (10.3)	20 (11.4)	1
Recurrent hospitalization, *n* (%)	5 (12.5)	17 (9.7)	0.5
In-hospital mortality, *n* (%)	1 (2.6)	30 (17)	0.037
One-year mortality, *n* (%)	2 (5.1)	22 (12.5)	0.2
Length of hospital stay, median (IQR)	31 (18;45)	27 (15;42)	0.08

## DISCUSSION

Right-sided endocarditis is less common than LSE. In this study, 18.1% of patients with IE presented with RSE, which was comparable to historical data.^1^,^2^ However, RSE is common among IVDU; 86% of IE cases among IVDU present with RSE.^21^ In our study 70% of RSE patients and 4% of LSE patients were IVDU. Another significant source of RSE is a pacemaker lead infection. In our study a significant number of patients had had a cardiac or another surgical intervention. Our data confirmed that pacemaker lead infection has become a significant risk factor for IE and specifically for RSE.

According to a previous publication, RSE patients are younger as compared to LSE patients.^22^ Our study confirmed this finding. The higher percentage of RSE patients with recurrent endocarditis as compared to the LSE group is probably due to the increased prevalence of IVDU in the former population.

The pathogeneses of RSE and LSE are known to be different.^15^,^16^ The literature describes several pathogenetic mechanisms that differentiate between the two types of IE: not only the primary contact of the injected substance with the tricuspid valve in RSE, but also intimal damage and thrombus formation, cocaine-induced endothelial damage with secondary pulmonary hypertension, valvular damage and infection, pressure gradient and turbulence,^15^,^22^,^23^ changes in the valvular endothelium,^24^ and different cytokine expression.^25^ In our study, RSE patients had a significantly more prominent CRP elevation. Elevations of CRP occur in association with acute and chronic inflammation due to a range of causes, including infectious diseases and non-infectious inflammatory disorders. Markedly elevated CRP levels are strongly associated with bacterial infection.^26^ Acute inflammation generally shows a marked CRP response, while low-grade inflammation shows only a minor CRP elevation. The inflammatory response to infection and tissue injury supports host defense, clearance of necrotic tissue, adaptation, and repair, while the purpose of low-grade inflammation appears to be restoration of metabolic homeostasis.^27^ The increased CRP levels found in RSE patients in our study may be due to their young age, resulting in a more prominent inflammatory response, and the respiratory injury, particularly pulmonary parenchyma, often seen in RSE patients as compared to LSE patients. This requires further investigation and research.

It is not surprising that for RSE patients, the majority of whom are IVDU, the percentage of patients with chronic liver diseases is higher, perhaps reflecting the higher rates of hepatitis B and hepatitis C infections and alcohol abuse in this population.

The most common pathogen in both groups of patients in our study was *Staphylococcus aureus*, consistent with previous data.^1^,^9^,^12^,^28^ It was found in more than half of the RSE group, as compared to less than 20% in the LSE group. The absence of coagulase-negative staphylococci in the RSE group was also notable in our study. These etiologic differences are important for management of patients with IE in our region.

The in-hospital mortality in our study was significantly lower in RSE patients as compared to LSE patients. One-year mortality was also lower in RSE groups compared to LSE groups, but did not reach statistical significance. These results are also similar to previous studies.^29^ It is known that the prognosis of RSE is better than for LSE, possibly due to the younger age of the RSE patients, and tricuspid valve involvement has few hemodynamic consequences compared to mitral valve dysfunction.^22^,^29^ The majority of RSE patients responded well to appropriate antibiotic therapy without complications and with no spread beyond the borders of the involved valve.^30^,^31^ Very few RSE patients need operative treatment of the involved valve.^21^,^32^–^34^ In our study only a minority of patients in both groups required operative treatment.

The results of this study have demonstrated a difference in the characteristics of RSE patients in our study versus previous studies, i.e. a documented higher percentage of patients with pacemaker lead infection and fewer who were IVDU. Indeed, intravenous drug abuse in Israel is less common. Pacemaker lead infection is the leading cause of RSE in Southern Israel. Due to the increased use of these devices, a high index of suspicion is needed so as to diagnose RSE at-risk patients with appropriate clinical presentations.

Another interesting finding of our study was that a mass attached to the electrode was just as common in LSE, indicating that sometimes left- and right-sided endocarditis can occur simultaneously.

The major limitations of this study are that it was a single-center investigation and that it used ICD codes to identify patients. However, patients were analyzed over a 10-year period.

## CONCLUSION

Our study demonstrated an increasing percentage of RSE compared to LSE among patients with IE in Southern Israel. This trend is expected to continue due to the increasing number of patients undergoing device insertion. Pacemaker lead infection has become the leading cause of RSE in Southern Israel; it is much less common to find RSE in IVDU. This study has also demonstrated etiological and clinical differences between RSE and LSE, with the RSE prognosis being better compared to LSE.

## Abbreviations

CRP: C-reactive protein
IE: infective endocarditis
IVDU: intravenous drug users
LSE: left-sided endocarditis
RSE: right-sided endocarditis
SD: standard deviation
SUMC: Soroka University Medical Center.

## References

[1] Frontera JA, Gradon JD (2000). Right-side endocarditis in injection drug users: review of proposed mechanisms of pathogenesis. Clin Infect Dis.

[2] Chan P, Ogilby JD, Segal B (1989). Tricuspid valve endocarditis. Am Heart J.

[3] Naidoo DP (1993). Right-sided endocarditis in the non-drug addict. Postgrad Med J.

[4] Nandakumar R, Raju G (1997). Isolated tricuspid valve endocarditis in nonaddicted patients: a diagnostic challenge. Am J Med Sci.

[5] Clifford CP, Eykyn SJ, Oakley CM (1994). Staphylococcal tricuspid valve endocarditis in patients with structurally normal hearts and no evidence of narcotic abuse. QJM.

[6] Yamashita S, Noma K, Kuwata G, Miyoshi K, Honaga K (2005). Infective endocarditis at the tricuspid valve following central venous catheterization. J Anesth.

[7] Michel PL, Acar J (1995). Native cardiac disease predisposing to infective endocarditis. Eur Heart J.

[8] Ortiz C, López J, García H (2014). Clinical classification and prognosis of isolated right-sided infective endocarditis. Medicine (Baltimore).

[9] Revilla A, López J, Villacorta E (2008). Isolated right-sided valvular endocarditis in non-intravenous drug users. Rev Esp Cardiol.

[10] Cremieux AC, Witchitz S, Malergue MC (1985). Clinical and echocardiographic observations in pulmonary valve endocarditis. Am Cardiol.

[11] Murdoch DR, Corey GR, Hoen B, International Collaboration on Endocarditis-Prospective Cohort Study (ICE-PCS) Investigators (2009). Clinical presentation, etiology, and outcome of infective endocarditis in the 21st century: the International Collaboration on Endocarditis-Prospective Cohort Study. Arch Intern Med.

[12] Haque NZ, Davis SL, Manierski CL (2007). Infective endocarditis caused by USA300 methicillin-resistant Staphylococcus aureus (MRSA). Int J Antimicrob Agents.

[13] Saydain G, Singh J, Dalal B, Yoo W, Levine DP (2010). Outcome of patients with injection drug use-associated endocarditis admitted to an intensive care unit. J Crit Care.

[14] Chambers HF, Morris DL, Täuber MG, Modin G (1987). Cocaine use and the risk for endocarditis in intravenous drug users. Ann Intern Med.

[15] Stein M (1990). Medical complications of 1 intravenous drug use. J Gen Intern Med.

[16] Mouhaffel AH, Madu EC, Satmary WA, Fraker TD (1995). Cardiovascular complications of cocaine. Chest.

[17] Muthukumaran CS, Govindaraj PR, Vettukattil J (2005). Testicular swelling with pneumonia and septicaemia: a rare presentation of right-sided endocarditis. Cardiol Young.

[18] Chahoud J, Sharif Yakan A, Saad H, Kanj SS (2016). Right-sided infective endocarditis and pulmonary infiltrates: an update. Cardiol Rev.

[19] Bonow RO, Carabello BA, Chatterjee K (2008). 2008 Focused update incorporated into the ACC/AHA 2006 guidelines for the management of patients with valvular heart disease: a report of the American College of Cardiology/American Heart Association Task Force on Practice Guidelines (Writing Committee to Revise the 1998 Guidelines for the Management of Patients With Valvular Heart Disease): endorsed by the Society of Cardiovascular Anesthesiologists, Society for Cardiovascular Angiography and Interventions, and Society of Thoracic Surgeons. Circulation.

[20] Li JS, Sexton DJ, Mick N (2000). Proposed modifications to the Duke criteria for the diagnosis of infective endocarditis. Clin Infect Dis.

[21] Levine D, Crane LR, Zervos MJ (1986). Bacteremia in narcotic addicts at the Detroit Medical Center. II. Infectious endocarditis: a prospective comparative study. Rev Infect Dis.

[22] Welton DE, Young JB, Gentry WO (1979). Recurrent infective endocarditis: analysis of predisposing factors and clinical features. Am J Med.

[23] Kauffmann RH, Thompson J, Valentijn RM, Daha MR, Van Es LA (1981). The clinical implications and the pathogenetic significance of circulating immune complexes in infective endocarditis. Am J Med.

[24] Sullam PM, Drake TA, Sande MA (1985). Pathogenesis of endocarditis. Am J Med.

[25] Husby G, Pierce PE, Williams RC (1975). Smooth muscle antibody in heroin addicts. Ann Intern Med.

[26] Volanakis JE (2001). Human C-reactive protein: expression, structure, and function. Mol Immunol.

[27] Chovatiya R, Medzhitov R (2014). Stress, inflammation, and defense of homeostasis. Mol Cell.

[28] Hecht SR, Berger M (1992). Right-sided endocarditis in intravenous drug users. Prognostic features in 102 episodes. Ann Intern Med.

[29] Kamaledeen A, Young C, Attia RQ (2012). What are the differences in outcomes between right-sided active infective endocarditis with and without left-sided infection?. Interact Cardiovasc Thorac Surg.

[30] Robbins MJ, Frater RW, Soeiro R, Frishman WH, Strom JA (1986). Influence of vegetation size on clinical outcome of right-sided infective endocarditis. Am J Med.

[31] Miro JM, del Rio A, Mestres CA (2003). Infective endocarditis and cardiac surgery in intravenous drug abusers and HIV-1 infected patients. Cardiol Clin.

[32] Akinosoglou K, Apostolakis E, Marangos M, Pasvol G (2013). Native valve right sided infective endocarditis. Eur J Intern Med.

[33] Vallabhajosyula S, Varma MD, Vallabhajosyula S, Vallabhajosyula S (2016). Right-sided infective endocarditis in an Indian intensive care unit. J Glob Infect Dis.

[34] Lee MR, Chang SA, Choi SH (2014). Clinical features of right-sided infective endocarditis occurring in non-drug users. J Korean Med Sci.

